# Bottom-up perspectives on hospital-wide patient flow – a multi-site qualitative study of solutions to organisational paradoxes

**DOI:** 10.1186/s12913-026-14214-w

**Published:** 2026-02-20

**Authors:** Philip Åhlin

**Affiliations:** Department of Technology Management and Economics, Chalmers University of Technology, Vera Sandbergs Allé 8, Göteborg, 412 96 Sweden

**Keywords:** Healthcare, Efficiency, Productivity, Throughput, Frontline professional, Hospital system, Improvement

## Abstract

**Background:**

As the demand for healthcare outpaces capacity, improving hospital productivity has become critical. Research suggests that hospital-wide improvements in patient flow can enhance efficiency but has largely neglected the insights of frontline healthcare professionals without managerial responsibilities. This article explores those professionals’ perspectives on enabling efficient patient flow across hospitals.

**Methods:**

Semi-structured interviews were conducted with 15 nurses and 15 physicians at six tertiary and secondary care hospitals in Sweden, followed by thematic analysis based on inductive reasoning to identify meaningful subjects and themes.

**Results:**

Analysis revealed seven paradoxes experienced by frontline healthcare professionals that are associated with hospitals’ efforts to enable efficient hospital-wide patient flow and linked to leadership, organisational design, routines, professional culture, and technology. Associated tensions intensify under operational stress and often lead to overtime or compromised care. Professionals emphasised the need for more aligned structures, clearer patient flow strategies, performance metrics that support the efficient transitions of patients, more centralised coordination, better adherence to standardised routines, and investment in IT tools to improve decision-making. Meanwhile, a gap in nurses’ understanding of patient flows and patient progression and their limited authority and mandates to advance patients highlights the need for stronger nurse–physician collaboration.

**Conclusions:**

Enhancing hospital-wide patient flow requires increased system-level coordination, better-aligned hospital structures, and improved operational planning. The solutions proposed by frontline professionals also largely align with previously identified managerial strategies for improved hospital-wide patient flow, which suggests a shared understanding that could be leveraged to drive meaningful change.

**Supplementary information:**

The online version contains supplementary material available at 10.1186/s12913-026-14214-w.

## Background

Healthcare systems worldwide face increasing pressure due to rising patient demand [1], chronic staffing shortages [2], and ever-higher healthcare expenditures as a percentage of national GDP [3], all of which limit hospitals’ ability to provide appropriate care at the right time [4]. To enhance hospital efficiency, research has highlighted the importance of improving patient flow, meaning the coordinated movement of patients through various stages of care, from admission to discharge, while ensuring the efficiency and quality of service [5]. Studies have additionally shown that focusing on the movement of patients through hospitals can improve patient flow make discharge processes more efficient, and reduce length of stay [5–8]. Beyond gains in efficiency, enhanced patient flow has been specifically linked to improved medical outcomes, patient safety, and overall satisfaction [9, 10]. Although several studies have also highlighted the need to take a hospital-wide view when seeking to improve patient flow [11–14], research on how to enable efficient hospital-wide patient flows has largely centred on the views of healthcare managers and managerial strategies, while paying little attention to the perspectives of frontline healthcare professionals who interact directly with patients throughout their hospital journey. That oversight seems to stem from the complexity of healthcare’s organisational structures and their entrenched occupational hierarchies, power dynamics, and communication barriers [15]. It also seems to be influenced by the traditionally top-down orientation of research in organisational development and operations management [16, 17].

Healthcare professionals, including nurses, physicians, and other clinical staff, play a crucial role in the daily management of patient flow. For that reason, their perspectives can provide valuable insights into the practical challenges entailed in improving the efficiency of patient flow and opportunities to do so [14]. Although much previous research, while focusing on managerial interventions, has sidelined those frontline views and experiences, those same views and experiences have been integral to the success of improvement projects [18–20] and can illuminate how strategies for enhanced flow should be implemented in practice. Beyond that, because hospitals are complex organisations with often competing departmental objectives [17, 21, 22], understanding what enables and what hinders effective patient flow from a healthcare professional’s standpoint is essential if improvements are to be sustainable [18, 23]. Therefore, this article examines the perspectives of healthcare professionals without managerial responsibilities on how to enable efficient hospital-wide patient flow. In particular, it investigates the factors that such professionals identify as barriers and solutions to improving patient flow across departments and along care pathways. By incorporating their insights, the article affords a more comprehensive understanding of challenges and solutions for hospital-wide patient flow, all to ultimately support the development of more effective, more contextually relevant strategies.

Healthcare organisations often function as “job shops” where autonomous departments provide specialised services to address patients’ specific medical issues [24, 25]. Depending on the severity of their condition and treatment needs, patients referred to a hospital may visit one or several specialised departments. Both within and across departments, patient flow is thus often viewed in terms of process throughput, usually with the goal of improving efficiency and productivity [5, 7, 26]. In turn, effectively managing that flow involves minimising process delays, with length of stay serving as a key metric of performance [5, 27]. However, many stakeholders in the care pathway prioritise their own departmental efficiency over broader hospital-wide coordination and, as a consequence, inadvertently create bottlenecks [28, 29]. One of the most visible, pressing consequences of such inefficiency is hospital overcrowding, particularly in emergency departments, where delays in inpatient transfers due to a lack of available beds have blocking effects. Such effects not only prolong wait times for those departments but may also result in patients being admitted to the incorrect ward, which could compromise the quality of care [4, 5, 9]. Overcrowding often stems from hospital occupancy rates that exceed capacity, which extends throughput times, increases length of stay, and contributes to staff burnout. Addressing those issues requires not only improving discharge planning and inpatient flow but also balancing competing pressures within hospitals—that is, increasing efficiency while maintaining individualised, high-quality care. Studies have shown that standardising routines and streamlining processes can enhance throughput but may also risk reducing professional discretion or compromising patient-centred care, both of which are concerns frequently raised by frontline staff [30, 31]. Those tensions are particularly visible in fast-paced, high-pressure settings such as emergency departments, where staff need to constantly balance clinical judgement with organisational demands. Recognising and grappling with that tension is essential to developing effective, context-sensitive flow strategies.

**Fig. 1:**
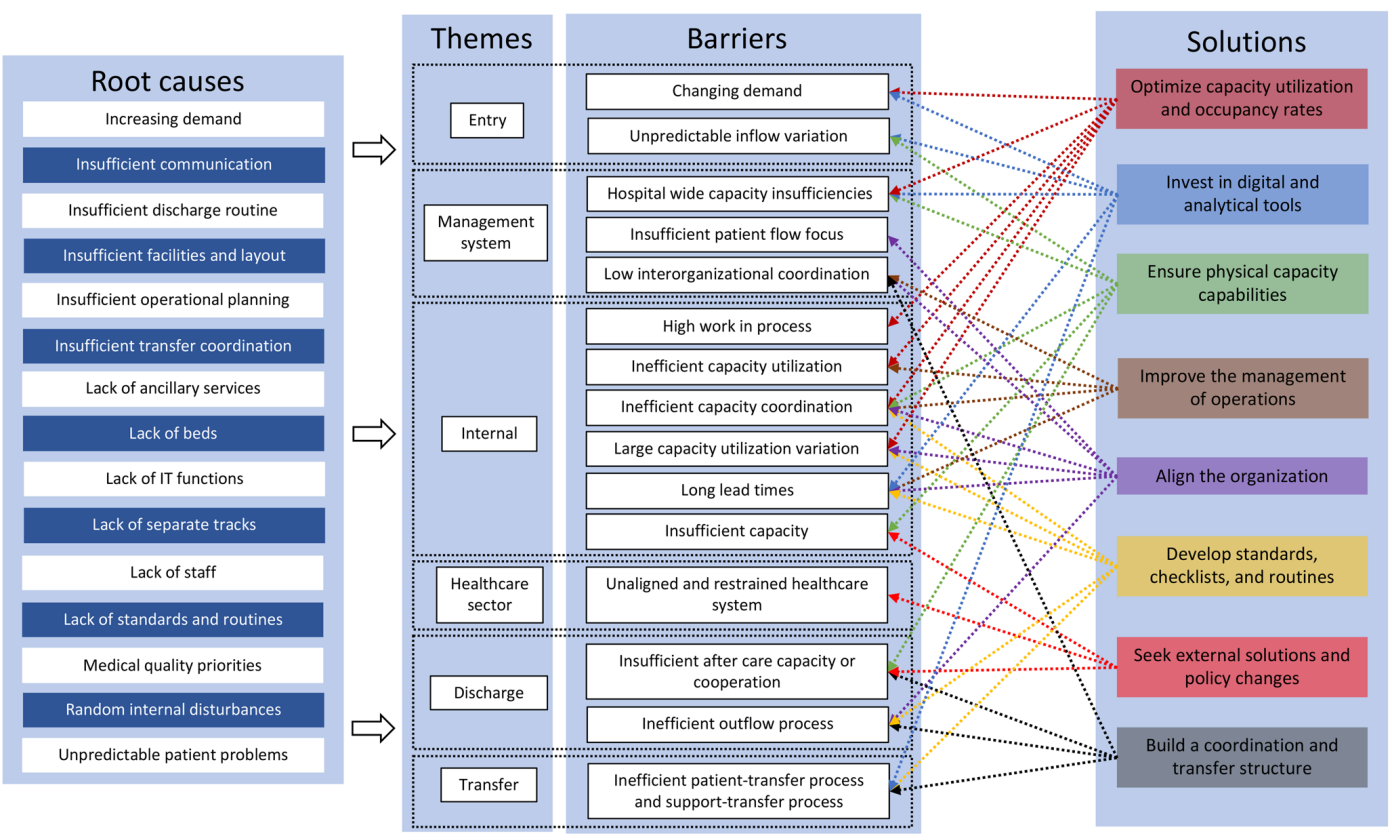
Hospital-wide patient flow improvement framework [13]

Among published frameworks that map barriers and suggest solutions for efficient hospital-wide patient flows, Åhlin et al. [32] have developed a taxonomy of hospital-wide flow challenges related to entry, transfer, internal processes, discharge, and the management system (Fig. 1). Their taxonomy highlights the importance for hospitals to align their organisations, build coordination and transfer structures, ensure physical capacity capabilities, invest in digital and analytical tools, improve their management of operations, optimise capacity utilisation and occupancy rates, seek external solutions and policy changes, and develop standards, checklists, and routines. Such models have contributed to current understandings of patient flow by systematically categorising process-related issues and potential interventions. Nevertheless, such approaches also often treat those issues as technical problems to be resolved through better coordination, standardisation, planning, or resource allocation.

However, as this article reveals, frontline healthcare professionals frequently describe challenges that are not merely logistical but deeply contradictory. In the study conducted for the article, participants voiced concerns about tensions between localised efficiency and hospital-wide collaboration, between professional autonomy and standardised protocols, and between short-term patient flow goals and long-term quality of care. Those tensions are not simply barriers to be removed but persistent organisational tensions that resist straightforward solutions. To better understand those dynamics, the study focused on paradoxes. Drawing on the theory of organisational paradoxes, particularly the framework proposed by Smith and Lewis [33], those tensions were conceptualised as “contradictory yet interdependent elements that exist simultaneously and persist over time”. From that perspective, the difficulties encountered in improving patient flow are not merely gaps in implementation but stem from competing demands that are embedded in the structure and logic of healthcare organisations themselves. In the study, taking that theoretical approach allowed moving beyond descriptive accounts of barriers and enablers to uncover how frontline staff navigate the simultaneous pressures of efficiency, quality, autonomy, and coordination. It also revealed why well-intended flow interventions may generate new problems that require staff to constantly adapt and reinterpret organisational goals in practice. As a result of the study, this article offers a more dynamic, grounded understanding of hospital-wide patient flow, one informed by the everyday experiences of the people most directly involved. By foregrounding paradoxes as a central conceptual lens, the article aims to provide insight into the complex realities of improving care coordination across hospital systems.

## Paradox theory

**Fig. 2:**
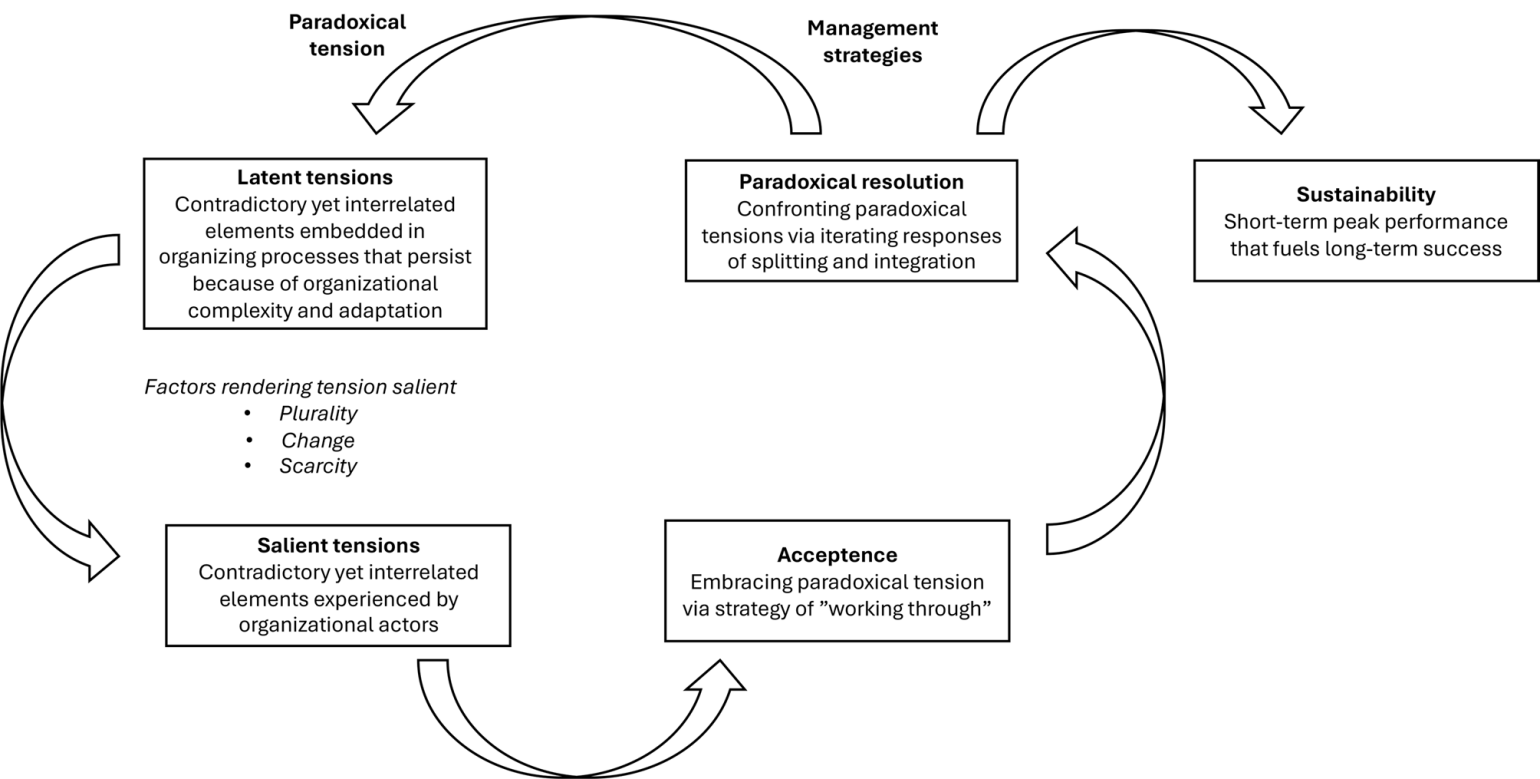
Model of organising in relation to paradoxes, adapted from Smith and Lewis [33]

Paradoxes or contradictions, as phenomena, are repeatedly encountered when leaders address fundamental questions on how to design and develop organisations and, in turn, establish boundaries that create distinctions and dichotomies [34]. In their influential paper, Smith and Lewis [33] write that leaders, in the process of forming organisations, “must decide what they are going to do, how they are going to do it, who will be responsible for it, and within what timeframe. By defining their objectives, leaders simultaneously establish what falls outside their scope”. That process clarifies strategic goals but also faces tensions, including global versus local priorities, social versus financial values, loose versus tight coupling, centralisation versus decentralisation, and flexibility versus control. Those tensions that emerge within organisations due to competing demands are paradoxes [35]—that is, “contradictory yet interdependent elements that exist simultaneously and endure over time” [33]. To promote the expansion of paradox theory, Smith and Lewis [33] also offer a dynamic equilibrium model of organising that can be applied (see Fig. 2).

The model underscores some key aspects of organising in relation to paradoxes. For one, paradoxical tensions within organisations exist both in latent and salient forms. For another, responses to those tensions involve cycling through different management approaches, and those approaches, in turn, have various impacts on sustainability. Beyond that, tensions may persist within organisational frameworks but remain dormant, unnoticed, or overlooked until external conditions or cognitive effort, as rendering factors, highlight their contradictory and interconnected nature. When latent tensions surface, they become more pronounced, and organisational members begin to directly experience their conflicting, inconsistent characteristics. Smith and Lewis [33] argue that rendering factors, specifically plurality, change, and scarcity, transform latent tensions into salient ones. Plurality arises with the coexistence of multiple perspectives in environments where power is dispersed [36]; such diversity amplifies uncertainty as objectives clash and processes become unaligned. Meanwhile, change introduces fresh opportunities for sensemaking as individuals navigate conflicting short- and long-term priorities [37] alongside competing yet interdependent roles and emotions. Last, scarcity pertains to constraints on resources, whether time, finances, or human capital. As leaders make decisions about allocating resources, those limitations intensify the struggle between competing yet interlinked alternatives [38]. Taken together, plurality, change, and scarcity push the boundaries of rational decision-making and strain organisational systems. As a consequence, individuals are more likely to fragment interconnected elements into either/or choices, actions, and interpretations and to obscure their inherent interdependence.

## Methods

### Design

The study followed a deductive methodological approach that involved using previous research as a foundation to extend the framework for efficient hospital-wide patient flows presented by Åhlin et al. [13] with new perspectives. The framework informed both the method of data collection and the analysis of the participants’ environment, challenges identified therein, and contextual factors. Added to that, a thematic analysis of the collected data was conducted that followed an inductive research approach to thoroughly explore the subjective perspectives of the participants, as recommended by Braun and Clarke [39] and Dixon and Woods [40]. The approach was chosen instead of predefining categories based on past research. To further interpret the complexity and inherent tensions identified in the data, paradox theory [33] was employed as an analytical lens; that theoretical perspective enabled a deeper understanding of how seemingly contradictory demands and dynamics coexist and shape hospital-wide patient flow. Last, the emerging themes from the thematic analysis, viewed through the lens of paradox theory, were integrated back into the framework to refine and expand its applicability.

### Data collection

In-depth, semi-structured interviews, conducted to collect the primary data, followed an interview guide (see Appendix) to ensure consistency across interviews while allowing participants the flexibility to freely share their perspectives [41]. The interview questions were designed to explore the barriers to and enablers of hospital-wide patient flow while remaining open to emergent themes and new insights. Hospitals were selected through purposeful sampling to ensure a diverse representation of healthcare professionals from both secondary care (i.e. services provided by medical specialists, often at hospitals, who in general do not have the first contact with patients) and tertiary care (i.e. highly specialised care delivered in a hospital or similar care setting) [42]. Six Swedish hospitals-three tertiary care providers and three secondary care providers—were included in the sample. Initial contact was made with the general directors of the selected hospitals, who were provided with information about the study’s purpose and asked to facilitate access to nurses and physicians actively engaged in managing patient flow. Nurses and physicians were chosen as the participants because they represent the two predominant healthcare professions most directly involved in patient flow. Upon receiving the invitation to participate, the general directors referred the research proposal to relevant department managers, who subsequently identified and facilitated contact with eligible participants. To capture perspectives from different hospital settings, healthcare managers were asked to recruit participants from emergency settings (i.e. emergency departments) surgical settings (i.e. surgical clinics, inpatient care units, and operating units), and medical settings (i.e. medical clinics and inpatient care units). Eligibility criteria required that participants (i) had no managerial responsibilities and (ii) had been employed and working in the same role for more than one year. Each hospital provided five participants, with one or two individuals from each setting, and 30 interviews with them were conducted between May and October 2023 (see Table 1). No participant chose to withdraw from the study. To ensure transparency, the interview guide was shared with all participants in advance. Interviews were conducted online via Zoom by a single researcher, with each session lasting 55–70 minutes.

**Table 1:** Participating hospital, type of hospital, hospital setting, clinic setting, and healthcare profession

Participating hospital	Hospital type	Hospital setting	Clinic setting	Healthcare profession	Participating hospital	Hospital type	Hospital setting	Clinic setting	Healthcare profession
A	Tertiary care	Emergency	ED	Nurse	D	Secondary care	Medicine	Clinic	Physician
		Surgery	Clinic	Physician			Surgery	Ward	Nurse
		Medicine	Clinic	Physician			Emergency	ED	Nurse
		Surgery	Ward	Nurse			Medicine	Ward	Nurse
		Emergency	ED	Physician			Surgery	Clinic	Physician
B	Tertiary care	Medicine	Clinic	Physician	E	Secondary care	Emergency	ED	Nurse
		Surgery	OR	Nurse			Surgery	Clinic	Physician
		Emergency	ED	Physician			Medicine	Clinic	Physician
		Medicine	Ward	Nurse			Emergency	ED	Physician
		Surgery	Clinic	Physician			Medicine	Ward	Nurse
C	Secondary care	Surgery	Clinic	Physician	F	Tertiary care	Medicine	Ward	Nurse
		Surgery	Ward	Nurse			Surgery	Clinic	Physician
		Emergency	ED	Physician			Emergency	ED	Nurse
		Medicine	Ward	Nurse			Medicine	Ward	Nurse
		Emergency	ED	Nurse			Emergency	ED	Physician

### Data analysis

All interviews were recorded and transcribed verbatim. During the interview process, detailed notes were taken, and initial observations of potential themes were documented. Afterwards, an open coding of the transcripts was performed that aimed at capturing the full range of perspectives amongst the healthcare professionals regarding factors that either hinder or facilitate hospital-wide patient flow. Each code was categorised as either a barrier or an enabler to ensure a comprehensive representation of viewpoints. The coding process followed an iterative and inductive approach, in which emerging themes were continuously refined to enhance categorisation and pattern recognition. The approach ensured that the analysis remained firmly grounded in the empirical data instead of being constrained by pre-existing frameworks. Once open coding was complete, codes were grouped into broader themes that move towards higher levels of abstraction. In the later stages of analysis, a consistent pattern emerged: many themes contained inherent contradictions. Participants frequently articulated ideal visions of how things should function while simultaneously describing lived experiences that starkly contradicted those ideals. Although they expressed values intended to guide healthcare delivery, the actual conditions often conflicted with those principles. Those observations highlighted the presence of paradoxical tensions within the data. To further explore those tensions, the dynamic model of organising paradoxes proposed by Smith and Lewis [33] was applied. Two aspects of the model were used in particular: the identification of emerging paradoxes and the strategies for addressing them. The third component of the model, cyclical responses to paradoxes, was deliberately excluded, because the study focused on identifying barriers and evaluating strategies for their resolution, not examining longitudinal shifts in organisational responses.

Next, the paradoxes embedded within previously identified barriers were examined more closely. The analysis explored how those tensions initially remained latent, what factors brought them to the surface, and how healthcare professionals proposed to resolve them. Participants’ descriptions of actions, structures, and strategies that mitigated those tensions or offered alternative approaches were coded and analysed. Those responses were often embedded within broader narratives about desired changes to improve patient flow. Codes related to “Suggested improvements” or “Desired changes” were clustered and interpreted in relation to the paradoxes identified. Last, the bottom-up solutions were mapped onto the existing framework for hospital-wide patient flow developed by Åhlin et al. [13], which provided a complement to the framework’s top-down structures. For a detailed overview of the analytical process and how the revised framework was developed, see Table 2.

**Table 2:** Overview of the analytical process and framework integration

Stage	Approach	Purpose	Description	Outcome/Emerging Themes
1. Interview design	Deductive	Ground data collection in existing knowledge	Interview guide developed using Åhlin et al.’s (2023) framework on hospital-wide patient flow	Interview guide
2. Initial coding	Inductive (open coding)	Capture participants’ lived experiences and perspectives	Verbatim transcripts coded without pre-set categories; codes labeled as barriers or enablers	650 open codes identified (e.g., “*Medical priority always takes precedence over logistical priority*” or “*the hospital is designed so that everyone only sees their small part of the patient flow*”)
3. Theme development	Inductive thematic analysis	Identify recurring patterns in the data	Codes clustered into broader themes using constant comparison	Themes such as: *“Responsibility and competence mismatch” or “Lack of hospital-wide understanding”*
4. Theoretical interpretation	Theory-driven (Paradox Theory)	Deepen understanding of tensions in themes	Themes re-analyzed using Smith & Lewis’ (2011) model of paradoxes (focusing on paradox emergence and paradox resolution)	Key paradoxes identified: *“Autonomy vs. Uniformity” or “Misaligned mandates”. Resolution paradoxes identified: “Build clearer flow strategy” or “Improved nurse flow mandate”*
5. Integration with framework	Deductive synthesis	Refine and expand existing framework	Emergent themes and paradoxes mapped back to Åhlin et al.’s (2023) framework to highlight gaps and new insights	The revised framework includes bottom-up solutions to complement previous top-down approaches to hospital-wide patient flow.

## Results

The study revealed seven paradoxes experienced by frontline healthcare professionals associated with hospitals’ efforts to enable efficient hospital-wide patient flow (see Table 3). The characteristics of each paradox are described in this section and supported by representative quotations from the interviews. In total, the interviews generated 650 unique opinions and recommendations, all of which were synthesised into the seven paradoxes. Table 3 presents a structured overview, beginning with a description of each paradox and the associated latent tensions. After that, it outlines the salient tensions that emerge and the rendering factors that make latent tensions salient.

**Table 3:** Seven paradoxes affecting efficient hospital-wide patient flow

Paradox		Latent tension	Salient tension	Rendering factor
**1**	*Local focus vs. system needs* *Unit-level loyalties conflict with system-wide flow coordination*	Collaborative issues	Overcrowding	Scarcity (Change) (Plurality)
		Unclear operational priorities	Clinic expansions	
		Ambiguis flow objectives		
**2**	*Fragmented expertise, whole patients* *Advocating for less rigidity, but specialisation builds rigidity*	Scheduling challenges	Overcrowding	Scarcity (Plurality)
		Slow patient transfer	Multimorbid patients	
		Operational inflexibility	Lack of OR capacity	
**3**	*Misaligned mandates* *Doctors hold authority, but nurses grasp patient flow*	Congestion consequence unawareness	Overcrowding	Scarcity Change
		Needing coordinating assistance	Long LoS patients	
		Slow patient progression	Unmet discharge planning	
**4**	*Seeking control, finding disorder* *Staff aim for workflow control but face constant disruptions*	Stressed doctors	Overcrowding	Scarcity
		Needing coordinating assistance	Unmet discharge planning	
		Deprioritization of flow	Long LoS patients	
**5**	*Autonomy vs. uniformity* *Compliance is expected, but autonomy often overrides routines*	Altered treatment plans	OR schedule overshooting	Plurality Change (Scarcity)
		Lack of long-term planning	Incomprehensible decisions	
		Unpredictable work days	Overcrowding	
**6**	*Proactive ideals, reactive reality* *Flow planning requires foresight, but care remains reactive*	Short planning horizon	Long LoS patients	Change Scarcity
		Little proactive planning	Unmet discharge planning	
		Stressful last-minute actions	Foreseeable avoidable bottlenecks	
**7**	*Data emphasis, flow blindness* *Statistical feedback abounds, yet neglects patient flow*	Low flow performance feed-back	New productivity requirements	Change
		Unmotivating metrics	Unsatisfying performance	
		Lack of best practice comparison		

### Paradox 1: Local focus versus system needs

#### Unit-level loyalties conflict with system-wide flow coordination

Healthcare staff across the studied hospitals described a persistent tension between local initiatives to improve patient flow and the lack of a coherent, hospital-wide flow strategy. Although some departments were reported to actively engage in identifying and addressing their own flow-related bottlenecks, many participants added that those efforts occur in isolation and without any shared framework or guidance from hospital management. The result is a fragmented approach in which each unit seeks to optimise its own operations, often at the expense of system-wide flow, and in which hospital management may discuss patient flow, but those discussions seldom transform into something tangible for frontline healthcare professionals:

*I imagine a bunch of managers having an idea of how patient flow should be done. But how is it communicated down? In my world, it doesn’t seem to be. It doesn’t seem to trickle down to all the different units but gets stuck somewhere along the way.* [Nurse, emergency department, tertiary care hospital, Hospital A]

The situation reveals a paradox in which individual departments are encouraged to take initiative and improve their own internal processes, but those localised efforts can inadvertently hinder broader coordination. When units develop flow routines tailored to their own needs, they often do so without accounting for dependencies on, or consequences for, adjacent departments. In doing so, they may create new bottlenecks, reinforce siloed practices, and/or resist taking responsibility for patients whose care trajectories do not neatly align with their own optimised processes:

*The general goal is for the flow of patients to go quickly but each department gets to develop its own routines. However, no one connects them across the hospital so that it’s really a working plan, put in place in different departments or different parts of the hospital, to really provide some improved flow. That’s what we would need.* [Physician (surgeon), surgical clinic, secondary care hospital, Hospital D]

That paradox becomes especially visible during periods of overcrowding or capacity strain, when the lack of central coordination leads departments to prioritise their own patients or avoid taking new ones, which only exacerbates problems with flow elsewhere. Efforts to improve flow at the unit level, albeit well-intentioned, can thus undercut system-wide performance, which reveals a core contradiction between the need for local control and the necessity of centralised coordination. Along those lines, participants expressed a desire for hospital management to resolve such tension by developing a clear, overarching flow strategy that aligns local initiatives with shared, cross-departmental goals. However, the same autonomy that empowers local problem-solving also entrenches siloed behaviour, which illustrates the difficulty of achieving both localised responsiveness and system-level integration—a hallmark of paradoxical demands in complex healthcare systems.

### Paradox 2: Fragmented expertise, whole patients

#### Advocating for less rigidity, but specialisation builds rigidity

Nurses and physicians often opposed the standardisation of healthcare processes by emphasising that the healthcare system has to remain responsive to each patient’s unique set of problems. They stressed the importance of individualised care, particularly in complex or multi-morbid cases. At the same time, many participants reported observing a strong, ongoing trend towards increased standardisation, largely driven by the growing specialisation of medical expertise and, to some extent, by changes in nursing practice. That shift is often justified by the belief that specialisation leads to higher-quality care through deep domain-specific knowledge and more efficient, repeatable routines. Participants noted, however, that patients increasingly present with multiple concurrent symptoms and diagnoses. In the past, such patients could more easily be categorised and assigned to the most appropriate department. Today, increased specialisation has made interdepartmental transfers more difficult, for departments are often reluctant to admit patients whose conditions fall outside their narrow areas of expertise. Such protectionism becomes particularly problematic during periods of hospital overcrowding, when the ability to move patients swiftly between departments is critical to maintain flow:

*Take, for example, an oncology patient with brain metastases quite far along in their disease who has palliative treatment in its final stages. They come into the emergency room with neurological problems and a headache, all signs that there’s a process in the brain. Then there can be a tug of war, or rather a push and pull, between the neurologist and oncologist about who should take care of the patient. The oncologist says that it’s an isolated neurological problem, while the neurologist says that it’s a complex oncological disease with an expected outcome that the oncologist can absolutely treat. But the oncologist is full, while the neurologist has one place left. It can be a discussion that can last for hours while the patient remains in the emergency room.* [Physician, emergency department, tertiary care hospital, Hospital F]

The quotation illustrates a core paradox experienced by healthcare professionals: while specialisation and standardisation are intended to enhance quality and efficiency, they simultaneously reduce the flexibility needed to care for patients with complex conditions. Thus, the very structures designed to streamline care for well-defined patient cohorts end up slowing care and creating bottlenecks amidst real-world clinical complexity. The same dynamic is present in surgical planning; surgeons are increasingly sub-specialising, which narrows the range of cases that they are willing or able to treat. As a result, the surgical schedule becomes highly rigid with minimal operational slack. Any fluctuation, including an unexpected staff absence or a surge in a particular patient group, can lead to cascading delays or cancelled procedures. Even so, there remains a strong professional belief that greater specialisation improves medical quality. However, that same specialisation requires greater standardisation and more homogeneous patient cohorts, which many healthcare professionals resist, particularly when treating diverse, acutely ill, or multi-morbid patients:

*The trend in the hospital, and healthcare in general, towards becoming increasingly specialised means that it’s a fairly fine-meshed net for patients to get through to get the care they need, and the flow of patients is slowing down.* [Physician, medical clinic, tertiary care hospital, Hospital B]

Therefore, the paradox lies in the tension between the push for standardisation through specialisation and the reality of increasingly complex patient needs. Instead of adapting care structures to fit patients, patients are expected to conform to increasingly rigid organisational logics. The shift reverses the ideal of patient-centred care and undermines both patient flow and the quality of care for patients who don’t “fit the mould”.

### Paradox 3: Misaligned mandates

#### Doctors hold authority, but nurses grasp patient flow

Healthcare professionals operate based on various underlying logics that influence their reasoning, work ethic, and areas of focus. A notable distinction frequently observed by both physicians and nurses was that nurses often possess a more intuitive understanding of patient flow than physicians. Nurses are typically trained to consider the patient’s entire care trajectory and to attend to the need for coordination in order to ensure continuous progression along the journey. By contrast, physicians tend to focus on the immediate clinical condition of each patient and make medical assessments and treatment decisions based on acuity, not the broader implications for overall system flow:

*There’s a lot to remember to do, and it feels very extensive. It’s very complex. At the same time, we’re faced with this problem that we cannot do everything with everyone. At the same time, we aren’t used to thinking about flow, either: “If I do this for this patient now, is someone else being displaced?” We aren’t trained in it that much.* [Physician, medical clinic, secondary care hospital, Hospital C]

The dynamic described gives rise to a fundamental paradox: although nurses often have a better grasp of patient flow and its operational implications, the legal and clinical authority to make decisions about patients’ progression lies solely with physicians, who may lack a flow-oriented perspective. As a consequence, responsibility and insight become misaligned. The professionals most attuned to optimising patients’ movement through the system are structurally unable to act on that knowledge, whereas the ones empowered to make decisions may inadvertently contribute to bottlenecks by focusing narrowly on clinical priorities. That paradox becomes particularly visible in inpatient wards, where less medically urgent patients may be deprioritised and left waiting for discharge decisions, often at the very end of the ward round. Nurses, despite playing a central role in coordinating patient logistics, are left to manage the downstream effects of delayed discharges and inefficient planning but without the formal authority to directly alter those plans. The result is growing frustration and inefficiencies in patient flow. Several participants emphasised the need for closer collaboration between professional groups and more integrated decision-making processes. Some also suggested that nurses should be granted greater authority to support and influence discharge planning and flow coordination.

Similar issues were described in the context of surgical scheduling, in which decisions made by surgeons are largely based on clinical urgency without always accounting for the impact on postoperative units or downstream bed availability. During periods of hospital overcrowding, those blind spots in flow awareness can significantly exacerbate congestion and delay care for others:

*The surgical programme could really be designed more according to the consequences it has on our departments. It feels like the surgeons only plan surgeries based on medical priority and very rarely based on what benefits the flow the most. Patients have much more predictable treatment times than many seem to believe, and thus the flow out could be much smoother.* [Nurse, medical ward, tertiary care hospital, Hospital A]

Thus, the paradox centres on a structural misalignment between authority and knowledge about flow: professionals with the best operational insight into patient movement lack decision-making power, while the ones with decision-making power often lack awareness of system-level consequences. Overcoming that paradox requires rethinking how responsibility is distributed and how collaboration is structured to better align expertise, authority, and accountability in the management of patient flow.

### Paradox 4: Seeking control, finding disorder

#### Staff aim for workflow control but face constant disruptions

Hospitals are frequently characterised as ranking amongst the most complex organisations in existence. They manage the simultaneous care of large numbers of patients with diverse needs, ranging from acute and semi-acute to scheduled and chronic conditions, while delivering advanced treatments that require coordination across multiple medical specialties and the integration of sophisticated technological and clinical systems. Considering such complexity, many physicians and nurses in the study underscored the substantial cognitive burden placed on senior doctors, who are expected to retain and process vast volumes of information each day to oversee the progression of all patients under their care. The challenge becomes particularly acute during periods of overcrowding, when numerous critical decisions need to be made concurrently under intense time pressure:

*I forget things, not because I’m careless, but there are many things to do at the same time. I have to focus on a task. In many cases, you don’t even have the opportunity to take notes. It can happen that I get a call, and I’m standing in a sterile gown operating or doing something. It means a lot of stress, in some cases an extremely high workload, and basically almost every day I feel unsatisfied with certain parts of my work. I’m fully aware that I don’t have time to do everything that needs to be done. I simply forget certain things.* [Physician (surgeon), surgical clinic, secondary care hospital, Hospital E]

The quotation underscores a core paradox in how work is organised in hospitals: although the complexity of care demands shared responsibility and distributed coordination, the system continues to concentrate information processing and decision-making on a few key individuals, most often senior physicians. As complexity increases, so does the expectation that individual clinicians will independently manage and remember an overwhelming number of details even though it is cognitively unsustainable. The substantial volume of information that has to be retained often results in critical details being overlooked, which requires nurses to devote considerable time to supporting physicians with prioritisation- and coordination-oriented tasks:

*Doctors sit and read beforehand but might not take into account whether the patient has anything else planned, and then it’s a bit of luck whether the patient is in the room or not. Quite a lot is up to me anyway, to keep track of different parts and sort of coordinate a bit so doctors don’t miss important things. Then, we also have social coordinators on the ward who help with planning care and such. And when they’re involved, they can be a good support. Either way, if we believe that a patient is going home on a certain date, then we have to think about preparing many things, and unfortunately, many things fall between the cracks.* [Nurse, medical ward, secondary care hospital, Hospital D]

Despite various efforts to introduce tools and roles to support coordination, the cognitive demands needed for such coordination remain disproportionately individualised. Under conditions of overload, professionals tend to prioritize the most critically ill patients, which increases the risk that patients with less acute conditions are neglected, particularly ones who remain hospitalised beyond their expected discharge dates. Both nurses and physicians underscored the negative consequences of concentrating too much responsibility on too few individuals and consistently called for a more equitable distribution of responsibilities. They additionally highlighted the need for system-level tools capable of visualising patient care processes and identifying next steps along the care trajectory. Such tools, they argued, could reduce reliance on individual memory and enhance workflow efficiency.

Thus, the paradox lies in the tension between the collective nature of hospital work and the persistent individualisation of responsibility. The system requires collaborative cognition and shared tracking of patient care, but simultaneously reinforces a structure in which key individuals are expected to “hold it all in their heads”—a practice that proves increasingly unsustainable under rising complexity and workload.

### Paradox 5: Autonomy vs. uniformity

#### Compliance is expected, but autonomy often overrides routines

Healthcare is a routine-driven sector in which the use of checklists and treatment protocols is intended to safeguard high standards of medical care. However, findings from the study reveal a paradoxical tension: while standardisation is widely promoted as a means to ensure predictability and efficiency, the culture of professional autonomy in healthcare actively resists such efforts. Most participants reported a lack of well-established routines and, even more commonly, a tendency amongst professionals, especially physicians, to deviate from existing protocols. Those deviations are not always perceived as errors but instead as expressions of clinical independence and responsibility:

*A physician decides something for a patient at the end of the week. Then another one comes along and says something completely different and changes the plans. The new physician wants to make her own opinion and assessment of the patient. It might take a day or two to do that. And then everyone starts with something new, some new thought. And then the next week comes along with some new doctor who again wants to form their own opinion about the patient. So I think we have a lot to work on there, on continuity.* [Nurse, medical ward, tertiary care hospital, Hospital B]

Such autonomy, while valued for enabling individualised care, also introduces significant unpredictability into the system. Colleagues often struggle to interpret shifting decisions, which can lead to frustration and redundancy. For example, treatment plans may be revised during shift changes such that examinations are repeated and/or hospital stays are extended, both of which hinder instead of help patient flow. Experienced professionals who work closely with patients often observed that those revisions rarely improve outcomes. Instead, many perceived that individual physicians prioritise their own judgement over shared protocols, even when existing guidelines are in place. The paradox becomes especially visible during periods of hospital overcrowding when coordinated action is essential. In those high-pressure situations, sudden, unilateral changes in plans of care can be deeply demotivating for staff and disrupt the flow of operations. A commonly cited example involves surgeons underestimating the duration of procedures despite repeated experience showing otherwise, which leads to operating room schedules being behind, staff working overtime, and increased variation in workload:

*Surgeons have been given operating blocks to deal with but plan surgeries so that they’ll be finished before the end of the block time even if they’ve never performed a surgery within the intended time. Then we have to work overtime. We usually realise that immediately when we see the schedule for the day. It doesn’t feel great that the original planning and staffing schedules aren’t respected. It creates a lot of variation, unpredictability, and overtime work for the unit.* [Nurse, operating room, tertiary care hospital, Hospital F]

Thus, the paradox lies in the conflict between the system’s need for standardisation to reduce variation, which is crucial for managing patient flow efficiently, and the deeply rooted norm of professional autonomy, which fosters variation and unpredictability. Although both sides aim to ensure high-quality care, they pull in opposite directions: one towards collective consistency, the other towards individual discretion.

### Paradox 6: Proactive ideals, reactive reality

#### Flow planning requires foresight, but care remains reactive

Healthcare operates within a dynamic environment in which patients’ conditions may improve or deteriorate rapidly, which requires corresponding adjustments in healthcare operations. Professionals recognise that plans often shift throughout the course of a single shift, both with respect to patient treatment and the optimal allocation of resources such as staff, treatment rooms, and equipment. In multiple interviews, a recurring concern was that the planning horizon is often too short, and greater foresight in operational planning was identified as a potential enabler of more efficient patient flow. Frustration was reported regarding cases in which patients remain in care longer than necessary simply because short-term planning prevents timely transitions or discharges:

*In the best of worlds, we would think more about the continuous patient trajectory, and I think it would be better if you already have a plan for the patient when they’re admitted. But unfortunately, it often happens that we only plan half a day in advance, until the next round, and after the round, and then until the following afternoon.* [Nurse, medical ward, secondary care hospital, Hospital C]

The quotation highlights a central paradox experienced by healthcare professionals: despite growing awareness of the value and feasibility of proactive, data-informed planning, daily practices remain entrenched in reactive routines. On one side of the paradox, the participating professionals expressed a strong belief that care trajectories can be predicted with relative certainty and that innovative technology can enable a more forward-looking approach. On the other side, existing structures, routines, and decision-making cultures were described as being optimised for short-term responsiveness and for planning from round to round, which constrains the ability to act proactively, even when predictable patterns are known. The professionals perceived hospital bed coordination as predominantly reactive and without a strategic role in optimising patient flow across the hospital or in planning patient movement with consideration for downstream effects. Many physicians and nurses also expressed the view that a more clearly defined coordination structure, endowed with greater authority, would support more efficient transfers between the emergency department, acute care units, intensive care units, and hospital wards. Although many bottlenecks are predictable, the absence of centralised coordination often results in recurring delays in patient progression, which several respondents described as disheartening. Interviewees added that patient care trajectories are more predictable than commonly assumed and suggested that a proactive, forward-looking approach to planning could significantly improve flow. Furthermore, new technology and/or innovative applications of existing technology were identified as potential tools to support healthcare professionals in adopting a more anticipatory strategy and enabling more accurate assessments of patient care trajectories through the application of statistical analysis:

*Patients come in clear prototypes, and you can determine with a fair amount of certainty how many days they’ll need to be cared for. And with new technology, we should be even better at determining when a patient is likely to be discharged. It allows us to be much more proactive in planning care than we often are, even several days before discharge, given everything that needs to be done.* [Physician (surgeon), operating room, secondary care hospital, Hospital D]

Thus, the paradox lies in the tension between the acknowledged potential for proactive planning and the deeply embedded reactive modes of operation. Despite having tools, data, and knowledge to plan ahead, the system remains stuck in short-termism: a cycle driven by immediate pressures, fragmented responsibilities, and an underdeveloped coordination structure.

### Paradox 7: Data emphasis, flow blindness

#### Statistical feedback abounds yet neglects patient flow

Improving performance requires an understanding of current performance levels. Although healthcare providers routinely engage in extensive measurement, particularly by continuously monitoring outcomes regarding medical quality, which is central to healthcare operations and development, the use of metrics related to patient flow appears inconsistent. When asked whether they utilise indicators reflecting patient flow performance, physicians and nurses offered varied responses. Some reported using flow-related metrics, including length of stay, average discharge time, and the average number of discharges before noon. Others, however, indicated that they do not employ any measures specifically linked to patient flow:

*No, we no longer get any information about whether we perform well or badly or how many patients we’re discharging at a certain time. I actually have no idea about anything related to patient flow. We get no feedback.* [Nurse, surgical ward, secondary care hospital, Hospital E]

A few interviewees reported receiving feedback from management, albeit infrequently. Notably, all participants working in units that employ flow metrics perceived those measures as lacking motivational value. Although the metrics themselves were not viewed as inherently problematic, the associated performance targets were frequently described as being unrealistic and largely unattainable. Consequently, participants expressed little motivation to engage with those measures and reported putting forth little effort to meet the prescribed targets:

*Even though there are a lot of things measured in association with surgical procedures related to patient flow, few or no one directly follows them or cares. The goals aren’t realistic, either. They’re simply not motivating. That’s a problem*. [Physician (anaesthesiologist), operating room, tertiary care hospital, Hospital F]

The situation described reflects a paradox: although staff are expected to take ownership of and contribute to improving patient flow, a key hospital performance goal, they are simultaneously deprived of the feedback and actionable data needed to guide or assess such improvement efforts. On the one hand, flow metrics are framed as being essential to efficient operations; on the other, healthcare professionals closest to the work are not systematically informed about those metrics or involved in interpreting them. That disconnect undermines professional agency and accountability, which creates a situation in which responsibility is assigned without providing the means for responsible action. Moreover, the consequences of failing to measure the performance of patient flow often become apparent when new managers or hospital leaders express dissatisfaction with production outcomes and introduce new performance targets or staff-to-patient ratios. In such cases, the unit may lack the necessary data to assess its current capabilities, determine the feasibility of the new requirements, and predict the impact of those changes on patient flow performance.

## Resolutions to paradoxes

**Fig. 3:**
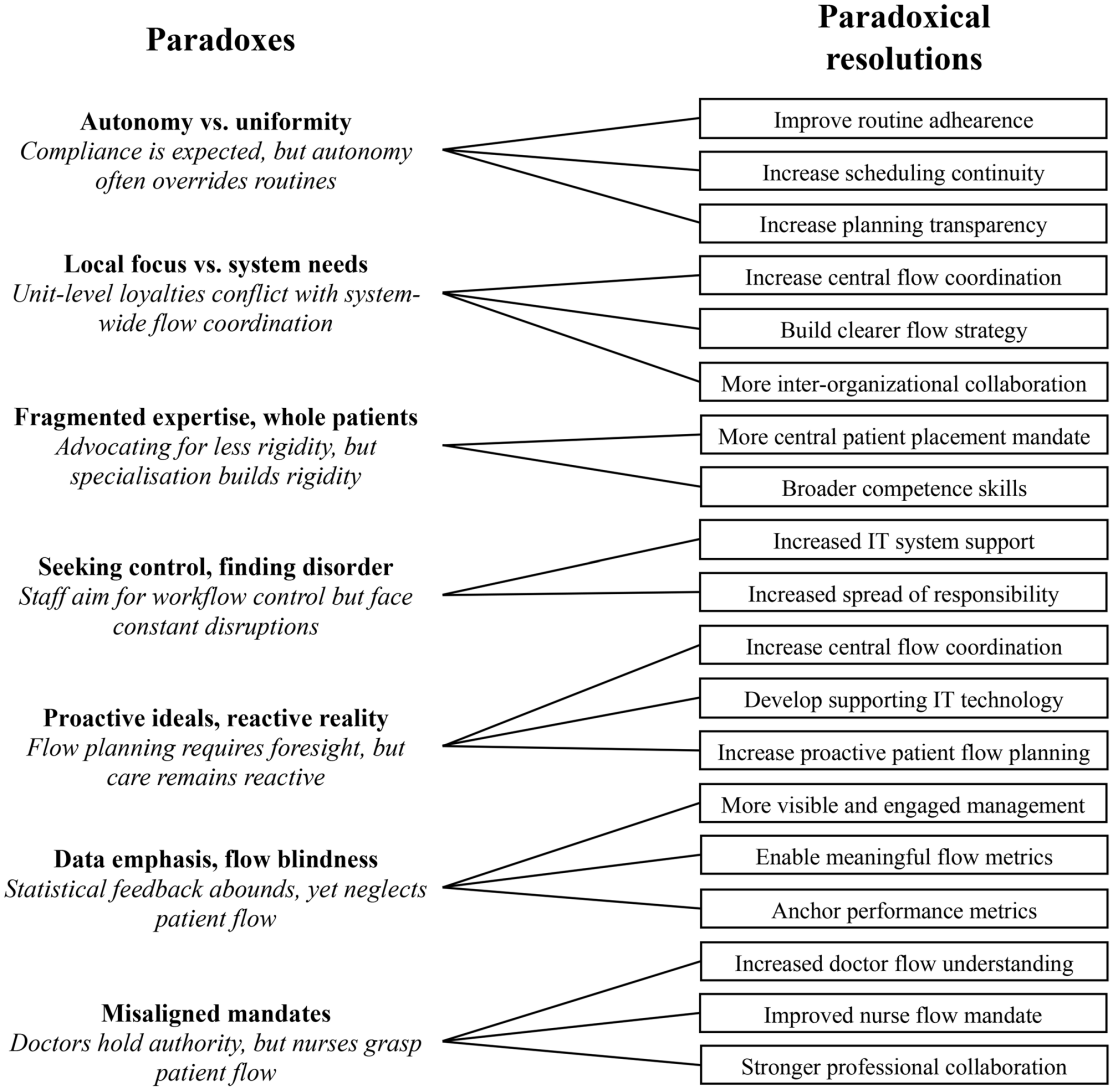
Paradoxes and resolutions to hospital-wide patient flows

The study revealed seven paradoxes that impede healthcare professionals’ efforts to improve patient flow across hospital systems. Although illuminating such paradoxes and contradictions in healthcare delivery is valuable, the initial aim of the research was to deepen the understanding of challenges in hospital-wide patient flow and explore potential solutions. Drawing on the perspectives of frontline healthcare professionals, this article presents a series of proposed resolutions to the identified paradoxes (see Fig. 3).

The resolutions are organised around the seven paradoxes. First, for improved patient flow, participants advocated stricter adherence to shared routines, improved continuity in staff scheduling, and greater transparency in planning processes. Those measures were viewed as fostering compliance and predictability in daily operations:

*We need to have much better consensus on the path that the hospital has chosen and the routines in place so that everyone sees the flow in the same way and understands it.* [Physician (surgeon), surgical clinic, secondary care hospital, Hospital E]

Second, participants called for the strengthened central coordination of patient flow, underpinned by a hospital-wide strategy and interorganisational collaboration. On that count, some respondents emphasised the need for a designated coordination function or team to ensure alignment across units:

*There needs to be someone, or a team of people, who connects the pieces to ensure that there’s a functioning plan for the different parts of the hospital: a plan that will truly lead to improved patient flow.* [Nurse, medical ward, tertiary care hospital, Hospital F]

Third, to reduce systemic rigidity, participants suggested expanding the competence profiles of staff. For example, requiring nurses and physicians to take on broader roles and competencies in parallel to their specialisation in a medical subfield was proposed as a way to improve operational flexibility. Others emphasised the need for a centralised mandate or authority, a flow coordinator or team, empowered to make real-time placement decisions.

Fourth, improved control over workflows was linked to both better IT support and more distributed responsibility amongst clinical staff. For instance, developing patient logistics systems that may aid clinicians in managing a pressing situation and better balancing decisions between what is important for both individual patients and the general patient flow:

*There needs to be a more automated system—a medical records system where you could see where in the chain the patient is, so that all parties can see the patient’s location, what’s supposed to happen next, and where the bottlenecks occur.* [Nurse, medical ward, secondary care hospital, Hospital D]

Fifth, a more proactive approach to care delivery, including earlier discharge planning and anticipatory bed management, was tied to stronger central oversight and supportive IT tools. Several participants described the need to shift from reactive to anticipatory planning:

*We need better forecasting tools for improved proactive planning of patient care trajectories. We need to be able to discuss earlier in the process whether things are possible or not. We need to know things together weeks in advance so that the surprises are reduced.* [Physician (anaesthesiologist), operating room, tertiary care hospital, Hospital F]

Sixth, participants stressed the importance of visible and engaged leadership. They called for the development of performance metrics that reflect actual flow dynamics and can be used meaningfully in daily work. Those metrics should be used not only for accountability but also for motivation and collective learning.

Seventh and last, to address professional silos, participants highlighted the need for the greater involvement of nurses in flow-related decisions and more regular interprofessional communication. Several also called for routine joint planning meetings or daily huddles involving both medical and nursing staff to promote a shared understanding of the dynamics of patient flow:

*Throughout the process, there are many, many parties involved, and the problem arises when you have to move from your own domain into someone else’s. That’s when it starts to become a bit problematic because you need help from someone else. That’s why we need to meet each other more, both informally and formally*. [Physician (surgeon), surgical clinic, secondary care hospital, Hospital D]

Altogether, those proposals represent a bottom-up perspective on resolving systemic tensions and paradoxes. Although some of the suggestions align with existing best practices (e.g. flow coordinators and IT systems for patient tracking), others reflect deeper organisational changes, including the redistribution of decision-making authority and redefinition of professional roles.

## Discussion

Improving patient flow at hospitals is crucial to meeting the growing demands of future healthcare, and prior research highlights how enhanced patient flow is a key factor in boosting hospitals’ productivity [5–8]. As patients increasingly navigate complex care pathways involving multiple professionals, departments, and administrative units, a system-wide perspective has become essential [11–14]. However, most studies on hospital-wide patient flow have focused on managerial perspectives and strategies while largely overlooking the experiences of frontline healthcare professionals—that is, the healthcare workers who interact most directly with patients throughout their hospital journey. Although healthcare professionals often articulate a clear idea for achieving efficient patient flow in their organisations, their experiences frequently diverge from those ideals. They uphold core organisational objectives and values but routinely encounter situations that contradict them. Based on such findings from participating healthcare professionals, the study conducted for this article revealed seven paradoxes associated with enabling efficient hospital-wide patient flows in which non-managerial staff at large hospitals perceive direct conflicts between the hospital’s stated values, philosophies, and objectives. Those contradictions contribute to a work environment characterised by frustration, stress, and unpredictability.

Paradoxes in patient flow have been previously studied by Kreindler [14], who examined systemic barriers by conducting interviews with a large number of hospital managers in a region of Canada. The study identified three key paradoxes [1]: “initiatives improve parts of the system but fail to address underlying systemic constraints” [2], “local innovation clashes with regional integration”, and most notably [3], “rules that improve service organisation for my patients create obstacles for yours”. A common theme amongst those paradoxes, as identified by Kreindler [14], is their emergence largely due to the absence of a system-wide approach to patient flow and its optimisation. Enhancements in one part of the system often create unintended challenges elsewhere, and such improvements may not align with the workflows of the broader system. The results of the study presented in this article confirm but also refine those paradoxes by emphasising that although autonomy benefits independent actors, it can disadvantage actors who depend on others. Similarly, while specialisation alleviates burdens for some, it often shifts the workload onto others.

The results additionally highlight the strong institutional belief in the capacities of physicians, which drives hospitals to assign them mandates and decision-making responsibilities that they may not be equipped to fully comprehend or manage. Delegating certain responsibilities of physicians to other professional groups appears to be advantageous for not only patient flow but also the professional development and effectiveness of physicians [43, 44]. Those paradoxes further underscore the perspectives of many healthcare professionals, who argue for designing workflows based on patient movement instead of the rigid structures of medical specialisations [6]. Many professionals also report the prevailing local focus on patient flow, coupled with a reactive instead of proactive organisational approach that is increasingly outdated, especially given technological advancements and emerging organisational models that support a more anticipatory healthcare system [13]. Beyond that, choosing not to provide meaningful feedback on the performance of patient flow to healthcare professionals, despite the centrality of medical quality in hospital performance, appears to be unwise. Patient flow directly impacts individual patients by facilitating faster transfers and reducing iatrogenic complications while also improving the healthcare system’s efficiency by increasing overall accessibility [10, 45].

The results also shed light on the tension between efficiency and quality of care, an issue that has been documented but remains under-acknowledged in efforts to implement patient flow improvements. As observed by Nugus and Braithwaite [31], the drive to streamline patient flow often competes with clinicians’ professional commitment to individualised, patient-centred care. Similarly, Benjamin [30] has highlighted how emergency nurses perceive flow initiatives and standardised procedures as sometimes undermining their capacity to provide quality care. Our findings echo the same concern: although frontline healthcare professionals frequently recommend the increased use of standardised routines and schedules, they also express resistance when such measures are perceived as being overly rigid or misaligned with patient needs. Such ambivalence suggests that standardisation, albeit essential for system coordination, needs to be balanced with professional discretion and context-sensitive judgement. A more nuanced approach to flow improvement may be required, especially one that recognises the legitimacy of frontline concerns and incorporates flexibility within standardised processes.

Most of the paradoxes identified stem from some form of scarcity, whether of beds, staff, space, or operating room time. Healthcare professionals frequently encounter resource constraints and often attribute inefficient patient flow to a lack of adequate resources. The emergence of bottlenecks is one of the most common indicators of systemic inefficiencies [7]. For instance, when auxiliary beds are required due to ward overcrowding, deficiencies become apparent. Similarly, when cases for the operating room consistently exceed scheduled capacity, which force staff to work overtime for consecutive days, the system’s limitations become unmistakable. Paradoxes also arise in response to change, whether through modifications in routines, new regulatory requirements, or the expansion or restructuring of services. People are generally resistant to change, particularly when it is perceived as being irrational or difficult to comprehend, which often brings paradoxes to the fore [37]. Additionally, some paradoxes emerge due to plurality, conflicting ideas, interests, and mandates when it comes to decision-making processes [36]. Those tensions become evident when there is disagreement over where to place multi-morbid patients or when a culture of professional autonomy leads to shifts in patient care trajectories with every change in staff. The study’s results, however, underscore that many of those paradoxes, whether driven by scarcity, change, or conflicting interests, can likely be mitigated through enhanced system-wide collaboration, a deeper understanding of patient flow dynamics, and a greater adherence to standardised routines and operational planning. Those findings also align with the results of past research advocating for reducing unnecessary variation in healthcare processes in order to improve overall efficiency [13]. Nevertheless, as the findings and the literature suggest, such adherence has to be sensitive to the perceived trade-offs between quality and efficiency. Without addressing those tensions directly, top-down flow-oriented initiatives may encounter resistance or be inconsistently implemented.

**Fig. 4:**
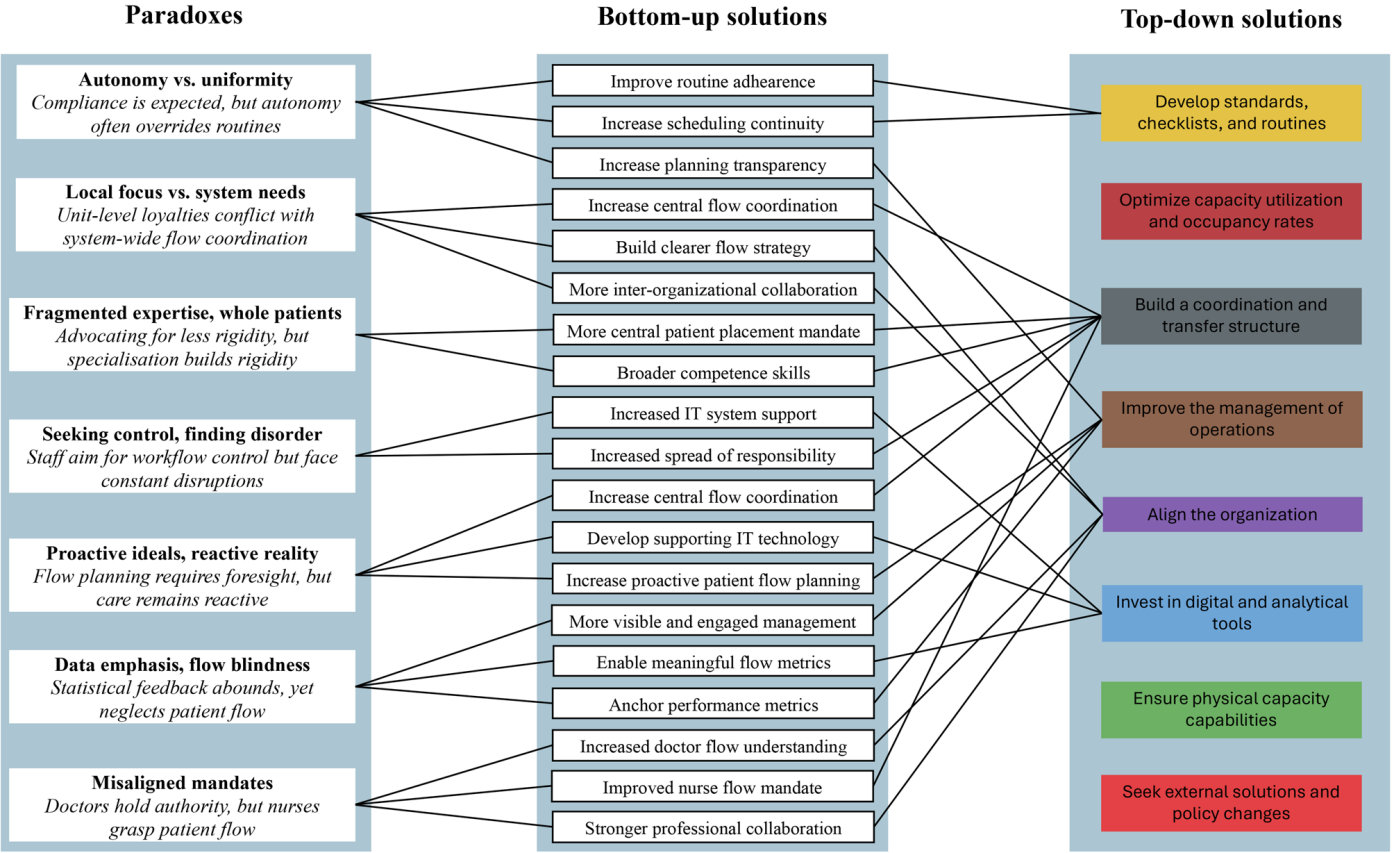
Comparison of bottom-up and top-down solutions to improve hospital-wide patient flow

The study adopted a bottom-up perspective that complements the patient flow improvement framework proposed by Åhlin et al. [13], which is grounded in the viewpoints of healthcare managers. The association between paradoxes and paradoxical resolutions presented in Fig. 3 has therefore been linked to the categories of solutions presented in the framework on hospital-wide patient flows (see Fig. 1) by Åhlin et al. [13] in a comparison of bottom-up and top-down solutions to improve hospital-wide patient flows (see Fig. 4).

The comparison shows that frontline healthcare professionals largely align with senior management in their views on how to enhance hospital-wide patient flow. Similar to senior leaders, frontline healthcare professionals emphasise the importance of developing standards, checklists, and routines, for they highlight the need to improve adherence to routines and set schedules. However, their support for standardisation is often contingent on whether those routines are perceived as enhancing, instead of constraining, their ability to deliver high-quality care. They also see the benefits of building a coordination and transfer structure, for they highlight the need for central flow coordination and placement mandates but also the need to expand and better use competencies amongst healthcare professionals to promote patient flow. Healthcare professionals also highlight the need to improve the management of operations by making production planning more transparent and proactive, using more meaningful flow metrics, and making the management more visible and engaged in improvements for patient flow. Aligning the organisation is viewed as being important due to enabling clearer flow strategies, increased system-wide understanding of the patient flow, and stronger collaboration between both professional groups and interorganisational units. Last, they also want to invest in digital and analytical tools to improve the support that healthcare professionals can receive from IT systems and to develop visible, better-synchronised flow metrics across the organisation.

Concerning the three categories without connections (i.e. optimise capacity utilisation and occupancy rates, ensure physical capacity capabilities, and seek external solutions and policy changes), no paradoxes or paradoxical resolutions seem to be associated. The reason is likely that they are associated with the factors that make latent paradoxes salient instead of imposing organisational paradoxes on the patient flow. According to Smith and Lewis [33], paradoxes evolve throughout the process of designing and forming organisations. In the study conducted for this article, healthcare professionals articulated an ideal vision of how to improve the flow of patients throughout their organisations while simultaneously describing experiences that starkly contrasted with those ideals. The three categories of top-down solutions without connections to the bottom-up solutions seem to be more connected to needed resources (e.g. staff, beds, rooms, and facilities) than work methods. Consequently, if those categories of solutions are met (i.e. when there is little resource scarcity), then the seven paradoxes revealed by the study may remain latent without becoming salient.

Although the study revealed several paradoxes and bottom-up solutions that align closely with top-down strategies, it is essential to recognise the inherent complexity and unpredictability of hospital operations. Large hospital organisations are composed of numerous departments, professional groups, and workflows, each with specialised roles and interdependencies. The fluid and dynamic nature of patient care, particularly when faced with emergencies, comorbidities, and fluctuating demand, makes full alignment across all units inherently challenging. Healthcare professionals may share an overarching understanding of what improves patient flow; however, the sheer scale and variability of daily operations often limit the consistent application of those ideals. Therefore, though system-wide strategies and collaboration are crucial, their implementation has to remain adaptive and sensitive to the decentralised, often fragmented realities of hospital work.

A novel contribution of the bottom-up perspective lies in its focus on how healthcare professionals might reconfigure their roles, competencies, and responsibilities to better support patient flow. A critical issue identified is the misalignment between nurses’ understanding of flow dynamics and their lack of authority to influence decisions about patients’ progression. There seem to be two paths ahead: either hospitals need to expand nurses’ mandates to advance patients along their care trajectories or enhanced collaboration between nurses and physicians becomes essential to ensure that flow-related knowledge is effectively translated into action, thereby improving overall patient flow.

## Limitations and directions for future research

The study’s findings have several limitations. First, they come from a single analyst. Although employing multiple coders would have been preferable, rigorous participant validation ensured the integration of diverse perspectives. Moreover, the author, having completed several courses on research methods and multiple research projects, has extensive training in employing qualitative research methods. Second, peer debriefing was employed to identify biases, challenge interpretations, and consider alternative explanations. A well-structured, semi-standardised interview guide was utilised, which facilitated a more consistent comparison across participants. Furthermore, the study’s robustness was enhanced by the substantial number of participants and the inclusion of six hospitals, which strengthen the validity and generalisability of the findings. A third limitation pertains to the online format of the interviews, all of which were conducted via Zoom. The virtual setting posed challenges in accurately capturing nonverbal cues such as body language and facial expressions, which may have constrained the comprehensive interpretation of participants’ responses. Last, the researcher’s background in the field of Operations Management introduced inherent biases and preconceptions. Such bias was reinforced by the researcher’s prior professional experience in a hospital setting and collaborating closely with doctors and nurses, which may have influenced the understanding of the participants’ challenges and perspectives.

The findings highlight several promising avenues for future research. One potential topic is to investigate differences in managing patient flow and its outcomes across hospitals, particularly in relation to the extent of authority and autonomy granted to nurses. Two other areas of interest concern how a hospital-wide focus on patient flow is operationalised at various managerial levels and how it can be tailored to be meaningful and motivating at each level of the organisation. Last, it would be valuable to explore the appropriate degree of independence and autonomy that healthcare professionals, especially physicians, should be granted. Although such autonomy is often regarded as being essential for efficient, responsive healthcare, it can also introduce confusion, inconsistency, and unpredictability in the delivery of care.

## Conclusions

This article examines the perspectives of frontline healthcare professionals regarding the factors that hinder and facilitate hospital-wide patient flow. Nurses and physicians reported experiencing significant contradictions between the ideals guiding how work should be conducted and the realities of how it is actually conducted. The analysis identified seven key paradoxes related to leadership and organisational structures, routines and procedures, professional culture, and the use of technology and performance metrics. Those paradoxes become particularly pronounced under organisational pressure, during changes to work routines, when patient care is compromised, or when staff are required to work overtime. In response, this article showcases multiple strategies aimed at addressing those paradoxes and enhancing the overall flow of patients throughout the hospital. Frontline healthcare professionals largely align with senior management on strategies to improve hospital-wide patient flow by emphasising the need for aligned structures, objectives, and performance metrics. They also endorse centralised coordination, the adherence to standardised routines, and responsive hospital designs. Importantly, professionals additionally highlighted the need to redefine roles and empower nurses to address the gap between their flow-related knowledge and limited decision-making authority. Strengthening nurse–physician collaboration is additionally essential to translating that knowledge into improved patient flow.

## Electronic supplementary material

Below is the link to the electronic supplementary material.

Supplementary Material 1

## Data Availability

All datasets generated or analysed during the study are included in the main article or its supplementary files.
