# Supplementary material for: Bottom-up perspectives on hospital-wide patient flow – a multi-site qualitative study of solutions to organisational paradoxes
Source: BMC Health Serv Res. 2026 Feb 20;26:306. doi: 10.1186/s12913-026-14214-w (PMC12937565; doi:10.1186/s12913-026-14214-w)
Supplement: Supplementary file 1 — Supplementary Material 1 [file 12913_2026_14214_MOESM1_ESM.pdf]

Chalmers University of Technology

Interview Guide

Hospital-wide patient flows

1.	Describe what you do in your professional role at the hospital
2.	Describe the function of your unit/clinic within the hospital
3.	Describe in a bit more detail what a typical week at the hospital looks like for you.
4.	How would you describe what a patient flow is?
5.	How would you describe how you and your unit/clinic work to improve your patient flow?
6.	Describe your view of how your and your unit's/clinic's work is related to the flow of patients through the hospital's overall organization.
7.	How would you describe an effective flow of patients through the hospital's entire organization?
8.	Who do you believe is responsible for improving the flow of patients through your hospital?
9.	What is your view on how your hospital currently works to enable a more effective patient flow throughout the organization?
11.	What have you and your unit/clinic done so far to improve patient flow in your hospital?
12.	What changes would you like to see within your unit's/clinic's and the hospital's organization to enable a more effective flow of patients through the hospital?
